# Food Oral Processing—An Industry Perspective

**DOI:** 10.3389/fnut.2021.634410

**Published:** 2021-02-09

**Authors:** Marine Devezeaux De Lavergne, Ashley K. Young, Jan Engmann, Christoph Hartmann

**Affiliations:** Nestlé Research Center, Lausanne, Switzerland

**Keywords:** biophysics, mechano-reception, taste molecular release, aroma release, sensory perception, age-appropriate products, nutrition for special medical purposes, biomimetic

## Abstract

We illustrate how scientific understanding of Food Oral Processing enables food product development with specific benefits for several target populations. *in vivo, in vitro*, and *in silico* approaches are discussed in the context of their ability to quantify oral processing from the molecular to the macroscopic scale. Based on this understanding, food structures with enhanced performance in terms of hedonic and nutritional properties as well as appropriateness for age and certain medical conditions can be developed. We also discuss current gaps and highlight development opportunities from an industry perspective.

## Introduction

**Figure 1:**
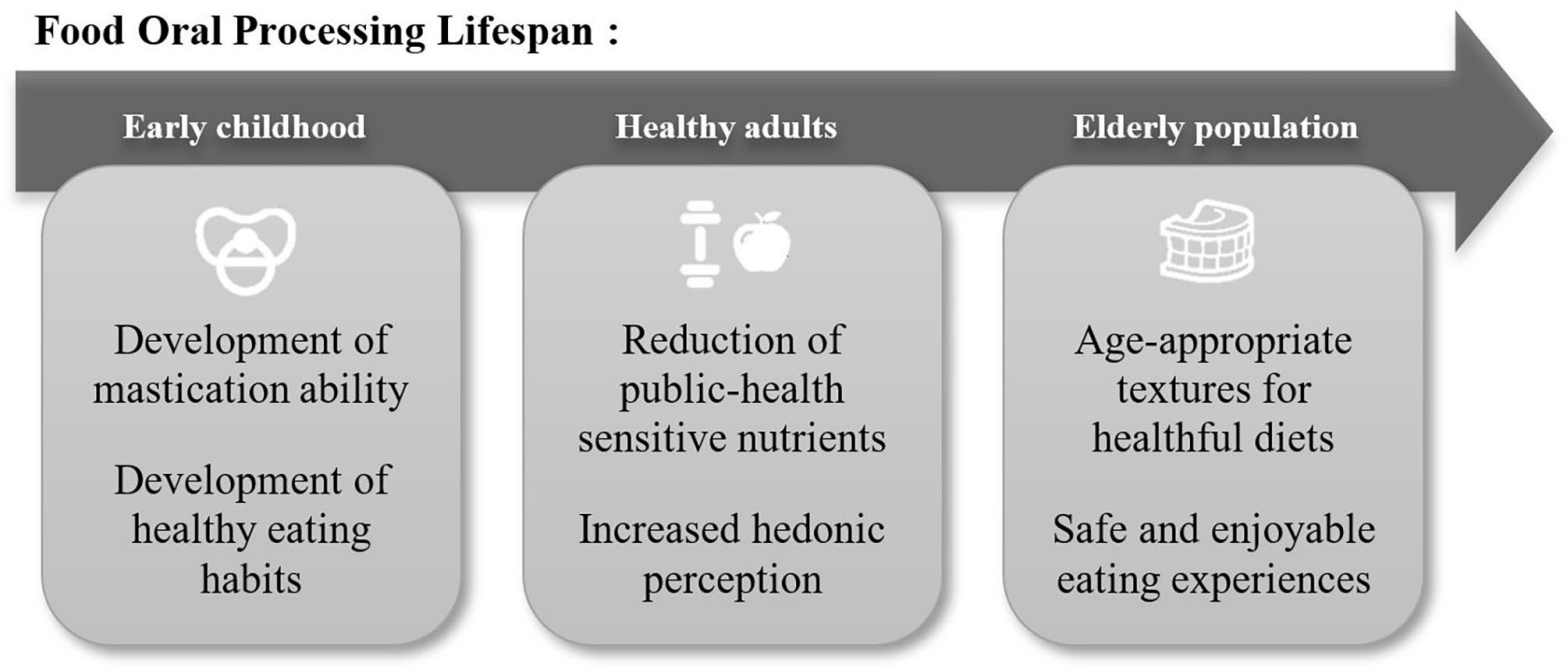
Food oral processing lifespan.

Food Oral Processing as the initial phase of food breakdown in the human body is critical from both nutritional and sensorial points of view. The central part of sensory perception is formed dynamically during oral processing (1–3). Understanding this breakdown process provides opportunities for food innovators to offer new sensory experiences to consumers (4). Furthermore, this is also the phase where the food is transformed into a swallowable and digestible bolus. The ability to break down food is a skill acquired in early childhood (5) which can be compromised under certain medical conditions such as dysphagia (6) at a later stage in life (Figure 1).

From an industry perspective, it is thus of utmost importance to understand food oral processing along several dimensions: consumer group (across the life span), food matrices (liquid, solid, degree of structure), and targets (sensory, behavioral, nutritional etc.).

In agreement with other studies in the field, those examples reveal great potential but also limitations of this emerging discipline. One important limitation for further translation into food industry applications are constraints related to experimentation in humans (sensory tasting, sampling of saliva, bolus, clinical trials) often requiring considerable recruitment, training, and scheduling effort/cost.

Given the complexity of physical processes and kinematics in oral processing, the need for a clear purpose when designing *in vitro* or computational (*in-silico*) models should be emphasized: what is the key output (target), e.g., residence time in the mouth, maximum bolus deformation? What are key control variables (e.g., speed of jaw movement)? Which influencing factors (e.g., dimensions of oral cavity) are assumed constant and with what justification? Mere “imitation” of biological processes guided by convenience of implementation can result in non-representative or even misleading results and should be avoided.

## Food Oral Processing to Stimulate Age-Appropriate Eating Habits

At birth, food oral processing starts with the suck-swallow-breathe reflex as the only mean for nutrition at the breast or the bottle. Infant oral movements during breastfeeding have been related to weight gain and self-regulation (7, 8), better oral-facial development (9, 10) and better acceptance of food textures (11) when compared to bottle-feeding. Milk flow is one of the parameters that differs between breastfeeding and bottle-feeding, and this flow can be modeled (12). It was found that, apart from suction force, the opening size at the tip of the bottle was the main parameter impacting milk flow in the bottle. However, not much congruence between manufacturers exists in labeling of baby bottle flow (13), thus we believe regulations on nipple flow would support development of bottle feeding closer to nature.

As a child grows, the tongue protrusion reflex fades and solid foods can be introduced. The mastication process matures as the muscles, bones, teeth, lips, and tongue develop (14). It has been reported that soft solids (purées, gelatin) can be efficiently masticated from 8 months of age, while harder solids (e.g., extruded cereals) are suitable from 24 months of age (5). Simione et al. (15) studied the chewing patterns of children between 9 and 36 months of age. They found coordination and motor control improved with increasing infant age. They hypothesized that chewing developed following two broad phases: the premolar (between 9 and 18-months) and molar (between 24 and 36-months) phases. Moreover, by studying the impact of food structures on infant eating patterns, they generated data that could inform science-based recommendations regarding the safety and appropriateness of foods. Designing textures for infant food products is key to achieving successful weaning and for establishing a solid basis for healthy nutritional habits, i.e., avoiding picky eating in toddlerhood (16).

## Food Oral Processing to Provide Healthy Pleasure

Consumers demand enjoyable tastes and textures. However, hedonic appreciation is often driven by health-sensitive nutrients (including sugars, salt, saturated fats) whose intake should generally be limited. Studying Food Oral Processing helps to understand physico-chemical and physiological parameters of sensory perception of such nutrients and leads to the design of food structures that deliver high sensorial quality with minimal use of undesired ingredients.

Food structure impacts *in vivo* aroma release. For example, modulating fluid viscosity impacts the release of retro-nasal aroma (17). In addition, modulating aroma release leverages sensory cross-modal interactions and enables reductions in public-health sensitive nutrients (e.g., salt) (18). However, oral processing parameters can outweigh the impact of mechanical properties of food, i.e., during *in vivo* aroma release from cheeses (19). Macroscopic structural characteristics can also impact aroma release. For example, the compatibility of the geometry of a piece of chocolate with the oral cavity imparts distinctive in-mouth melting patterns and enhanced flavor release (4).

In parallel to aroma release, oral food breakdown impacts the release, dissolution and diffusion of tastants before they reach taste receptors. Concerning beverages, a first strategy is to impact nutrient-sensing by modulating liquid microstructure and physical properties, such as low shear viscosity modulation (20) or use of emulsion droplets as a filler (21). A second strategy to lower nutrient concentration is heterogeneous distribution. In liquids, taste enhancement by pulsatile stimulation of taste receptors has been evidenced using gustometers (22, 23) and can be applied to products through smart packaging design (24). In solid products, macroscopic spatial distribution allowed salt and sugar reduction (25, 26).

Designing mechanical texture of foods or beverages can be as complex as designing taste and aroma since relating product mouthfeel to microstructural and rheological properties is a considerable challenge. The flows during oral sensing are complex, products evolve in the mouth, e.g., by mixing with saliva. Different sensorial pathways (gustatory, trigeminal, mechanical and even visual) all play a role in the perception of attributes like “body,” “smoothness” or “creaminess” (27). In addition to refinement and extension of rheological characterization into the non-linear domain, there has been an increasing focus on behavior of thin films and “tribological” approaches (28–38). This approach has in some cases shown new correlations between *in vitro* lubrication and sensorial attributes. One should note, however, that classical tribometry yields primarily “friction factors,” i.e., energy dissipation between solid surfaces lubricated by a liquid whereas “oral tribology” is also concerned with perceived “roughness,” which is more related to local force fluctuations than total dissipated energy (39). Such fluctuations across oral surfaces should be considered in “oral tribology.” In this context, biological roughness and compliance of specific structures (papillae) on oral surfaces play an important role in mechano-reception (40).

## Food Oral Processing to Support Healthy Aging

Above ~45 years, nutritional needs and abilities to sense and orally process food and beverages typically evolve again (41, 42). Eating habits developed during earlier life stages should continue to be favorable for health and well-being. However, decreasing basal metabolic rates (43, 44) require lower energy intake and adaptation of eating habits (portion size, meal frequency, diet caloric density) does not occur “automatically.” Specific morbidities, e.g., reduced sensation (higher perception thresholds, e.g., for sweetness), may create further challenges. Lack of specific nutrients due to lower food intake can negatively impact health and create a “vicious cycle” of lower appetite and malnutrition.

The ability to safely and efficiently prepare food for swallowing and swallow can also decline with age. Such challenges are referred to as “dysphagia.” Estimates of its prevalence in specific age groups vary, but can be as high as 50% in elderly care facilities (45–47)^1^. This leads to nutritional, psychological and social burdens (48). Swallowing dysfunctions are frequently caused by neurodegenerative diseases (Alzheimer, Parkison) or severe health events such as cerebrovascular accidents (“stroke”) that are more prevalent at older age. Loss of dentition also reduces the ability to masticate food and swallow effectively.

The impact of dysphagia on dietary intake ranges from gradual (avoidance of specific foods) to severe (inability to swallow safely). In extreme cases this leaves surgical interventions or tube feeding as the sole remaining options. In less extreme cases, therapeutic interventions (training “swallowing maneuvers”) and modification of *food* (softening, particle size reduction) and *beverages* (viscosity increase) enable subjects to enjoy a wide diversity of diets in terms of nutrient intake and sensorial quality. Such modifications require mechanistic understanding of oral processing and swallowing to optimally design rheological characteristics (31, 49, 50).

## Discussion

From this analysis it becomes obvious that thorough understanding of food oral processing leads to better products with improved benefits for consumers. However, the number and quality of studies in this area suggest that fast transfer into industry application is hard to achieve, especially given the fact that R&D capacities are very heterogeneously distributed in the food industry.

Investigation of food oral processing *in vivo* will remain crucial, as inter- and intra-individual aspects are very important for advancing understanding of food oral processing and its impact on sensory, liking, food choice, and eating habits (51, 52).

However, from an industry perspective, product design builds on food oral processing insights based on averages across a consumer group until a paradigm shift toward personalized nutrition becomes reality. Hence, we first review *in vivo* methodologies, then move on to discussing *in vitro* and *in silico* alternatives as enablers of translation into industrial practice.

### Monitoring *in vivo*

Eating patterns can be measured using electromyography (EMG) and kinematics of jaw movements (KJM). For EMG, non-invasive surface electrodes can monitor the activity of superficial muscles involved in oral processing. The use of EMG for eating studies has been extensively reviewed (53–56). Of particular note is the review by Vinyard and Fiszman (57) which states that physiological research indicates EMG provides information regarding muscle activity and relative recruitment levels but cannot provide reliable estimates of absolute force and mechanical work. KJM methods consist of either a marker or transducer that is physically attached to the teeth (58, 59), or skin surface markers (or features) that track movement of the chin or other facial features (60, 61). Markers attached to the teeth have been found to significantly influence natural chewing behavior (62). The use of skin markers are less intrusive and set-up can be faster making this approach attractive for studies targeting specific consumer groups such as children or consumers with dysphagia. Simple 2D video jaw tracking of a sticker has been shown to provide similar oral processing parameter values to a 3D electromagnetic system for consumption of solid gels (61). Video recordings can generate a heavy data load that requires tedious analyses by researchers or semi-automated analyses using software but recent developments in AI are expected to alleviate this burden. Mathis et al. (63) for example, demonstrate how pose estimation from simple markerless videography, based on transfer learning with deep neural networks, can be achieved for various body parts in multiple species across a broad collection of behaviors. Overall, using video recordings exclusively may sacrifice accuracy but due to the speed and ease of implementation (61), 2D video recordings are becoming more widely used (52).

In parallel to mastication studies, the food bolus (spit-out) can be collected allowing *ex vivo* observation of the properties of foods that have been manipulated in the mouth. A wide range of physical and chemical characterization of the food bolus can be performed, such as particle size, and mechanical properties (64). Bolus properties have been successfully linked to taste and texture perception (65–67). However, there is still a lack of alignment in methods used to characterize boluses in literature. In addition to the analysis method, oral status of participants, number of chews and food properties must be controlled to allow inter-study comparison (68).

Clinical investigation of dysphagia often uses time-resolved X-ray imaging (“videofluoroscopy”) for objective and categorical characterization of swallowing (69, 70). Boluses need to contain contrast material (e.g., BaSO_4_) to distinguish bolus and physiological structures and follow their movements. As such techniques present some exposure to ionizing radiation, they are generally restricted to subjects clinically indicated for diagnosis and are not used with healthy subjects. Recruiting sufficient eligible subjects to derive robust conclusions is hence both costly and time consuming. To avoid exposure to radiation, ultrasound can be used as a safe, non-invasive method to investigate swallowing *in vivo* (71), for instance with infants (72). Ultrasound can be used with a variety of foods (73) with minimal impact on eating patterns. However, image analysis from ultrasound measurements is laborious and there is a lack of alignment in methodology across oral behavior studies. Moreover, the low image resolution and the limited field of view does not allow a full analysis of all oral and bolus movements.

Compared to sensory, additional barriers for industry use of human food oral processing studies include: few standardized methodologies; some methodologies only allow for data collection one participant at a time; expectorated samples require immediate analysis or protocols to limit degradation during storage; separate expectorated samples are often required for each analysis type and oral processing stage of interest; more-involved data processing and analysis.

### Biomimetic Devices

Major benefits of alternative approaches include product development acceleration by rapid testing of food prototypes, the obsolescence of ethical approvals, the possibility to iteratively and specifically tune experimentation toward certain aspects of investigation or to mimic different consumer groups.

Simple “oral de-structuration” steps have been carried out in literature; e.g., controlled mixing with saliva for liquids, mincing/grinding for solids (74, 75). To lubricate these *in vitro* boluses, adding sampled human saliva is the most representative solution, but a variety of artificial salivas have also been used. Enzymatic digestion of starches can be mimicked using artificial saliva containing minerals and human salivary alpha-amylase, as proposed in the international consensus for *in-vitro* digestion (76). In addition, mucins from the porcine digestive system or submaxillary bovine mucins have been used to reproduce the lubrication properties of human saliva (77). However, complex chemical interactions cannot be reproduced using artificial saliva with simplified compositions. For example, complexation with astringent compounds involves a variety of small and large salivary proteins, and these mechanisms are still yet to be fully described (78). In order to study the interaction of food compounds with the oral mucosa *in vitro* models of the salivary pellicle have been developed using human saliva (79, 80).

Artificial masticators and swallowing robots have been developed to more closely mimic Food Oral Processing (74, 81–83). Of the masticators reviewed the two most advanced devices with regards to studying oral processing are the Artificial Masticatory Advanced Machine (AM^2^) and the Chewing Simulator. The AM^2^ has been validated for a wider range of food types through comparisons with *in vivo* bolus particle size distribution (84–86). Whereas, the main advantage of the Chewing Simulator over the AM^2^ is the on-line monitoring of volatile aromatic compound release (87–89). Currently missing from these systems are the simulation of the more complex roles of the tongue in oral processing including its interactions with products being consumed. Recent advances that could be incorporated into future systems include the development of a soft robotic tongue for studying *in vitro* swallowing systems (90) and 3D-printed soft biomimetic surfaces designed to replicate tongue topography, wettability, and tribological performance (91).

In addition to masticatory robots, swallowing robots are an emerging field of research. Reviews on such swallowing robots (92, 93) noted that despite the development of a range of devices, there is not yet one device capable of mimicking the entire deglutition process throughout the oral, pharyngeal and esophageal phases. It is important to note that these artificial masticators cannot replace human studies which are still required for: (1) system validation using particle size distribution (82, 87); and (2) identification of masticator inputs such as forces, salivary flow rate, and chewing time and frequency (89).

We expect though, that with increasing amount of data from human studies, the parametrization of such robots will be more practicable and therefore a dramatic gain in flexibility for future experiments is expected. It will thus become possible to design structures for a broader range of products with lower experimental effort and by leveraging learnings between studies more easily.

### Numerical Simulation

Experimental approaches to understand food oral processing have been complemented by mathematical modeling and simulation. These approaches usually focus on an isolated aspect of food oral processing, e.g., resolution limits for detection of solid objects (94, 95), fracture mechanics [e.g., (96)], effects of friction and wear [e.g., (97)], heat transfer and melting [e.g., (4)] or swallowing (50).

Integration of numerical simulation methodologies across scales (from supra-molecular to the continuum scale) and across governing physical principles and equations (molecular dynamics, fluid dynamics, heat and mass transfer, solid mechanics) has not yet been attempted. Recent work employing Lagangrian (particle-based) models bear the potential to integrate across multiple physical phenomena (98, 99). However, further integration of multi-scale approaches is required to produce predictive *in silico* approaches that ultimately could support the food product design process.

In future research, deep learning methods may help to integrate experimental techniques, data from biomimetic devices, *in vivo* approaches and computational approaches.

Similarly to our insights on biomimetic devices, we believe that *in-silico* approaches, validated thoroughly with *in-vivo* data, and in combination with *in vitro* tools, will allow inherently coupled processes to be addressed in a single study simultaneously; for example, the quantification of structure breakdown, tastant and aroma release, mixing with saliva. This would be impossible for *in vivo* studies because of the invasiveness of the quantification methods.

## Way Forward

We consider the increasing demand for plant-based products (100) in various consumer groups (young adults, adults, parents, patients, seniors) as one key driver of research in the area of food oral processing as chewing abilities vary across the life span.

For plant-based meat alternatives, for example, scientific understanding for the creation of meat-like textures will determine market penetration. Yet, an additional challenge is the creation of authentic meat-like taste and aroma experiences. Scientific understanding of tastant and aroma release during the oral process is crucial, as it depends inherently on food structure, mastication performance, and thus on the trajectory of oral food breakdown from initial structure to swallowable bolus.

Many plant-based dairy alternatives exhibit dry-mouthfeel, chalkiness and astringency induced by low-molecular weight compounds but also by proteins (101). In many products these defects are currently masked through flavorings, sweeteners, fats and hydrocolloids. Nutritionally more responsible products will gain consumer acceptance only if these defects can be solved through proper understanding of food oral processing.

In this manuscript, we focus on individual products. However, we would also like to highlight recent contributions addressing food oral processing across a whole meal or even diet (102, 103). Just like heterogeneous structures in an individual product, the spatio-temporal arrangement of different meal components modulates oral processing and thus impacts hedonic (e.g., liking) or health related (e.g., intake) outcomes. As these considerations require even more complex experimental arrangements with increasing permutations of food items (products in a meal) and their textures, we expect a strong demand for more flexible and robust methods also arising from this research.

We therefore pledge for an increased research intensity toward integration of currently co-existing scientific disciplines (food science, physiology, engineering). In the short to mid-term, a portfolio of biomimetic laboratory methods mimicking food-oral-processing from micro- to macro-scale, from comminution to swallowing and from solid to liquid matrices is required to develop new food solutions. This should be backed by faster experimentation methods on humans, and complemented with further developed *in silico* approaches.

## Data Availability Statement

The original contributions presented in the study are included in the article/supplementary material, further inquiries can be directed to the corresponding author/s.

## Author Contributions

CH initiated the work on this manuscript and brought the team of authors together. MD coordinated the writing and submission process. All authors contributed equally to the redaction and the content of this manuscript.

## Conflict of Interest

The authors declare that the research was conducted in the absence of any commercial or financial relationships that could be construed as a potential conflict of interest.

## Footnotes

^1^Prevalence in the general population is difficult to assess due to lack of systematic screening and unified terminology.

## References

[1] Lenfant F Loret C Pineau N Hartmann C Martin N. Perception of oral food breakdown. The concept of sensory trajectory. Appetite. (2009) 52:659–67. 10.1016/j.appet.2009.03.003 19501764

[2] Foster KD Grigor JMV Cheong JN Yoo MJY Bronlund JE Morgenstern MP. The role of oral processing in dynamic sensory perception. J Food Sci. (2011) 76:49–61. 10.1111/j.1750-3841.2010.02029.x 21535784

[3] Cheong JN Foster KD Morgenstern MP Grigor JMV Bronlund JE Hutchings SC The application of temporal dominance of sensations (TDS) for oral processing studies: an initial investigation. J Text Stud. (2014) 45:409–19. 10.1111/jtxs.12091

[4] Lenfant F Hartmann C Watzke B Breton O Loret C Martin N Impact of the shape on sensory properties of individual dark chocolate pieces. LWT. (2013) 51:545–52. 10.1016/j.lwt.2012.11.001

[5] Le Révérend BJD Edelson LR Loret C. Anatomical, functional, physiological and behavioural aspects of the development of mastication in early childhood. Br J Nutr. (2014) 111:403–14. 10.1017/S0007114513002699 24063732 PMC3927374

[6] Loret C. Using sensory properties of food to trigger swallowing: a review. Crit Rev Food Sci Nutr. (2015) 55:140–5. 10.1080/10408398.2011.649810 24915399 PMC4151816

[7] Li R Magadia J Fein SB Grummer-Strawn LM. Do infants fed from bottles lack self-regulation of milk intake compared with directly breastfed infants? Pediatrics. (2010) 125:e1386–93. 10.1542/peds.2009-2549 20457676

[8] Li R Magadia J Fein SB Grummer-Strawn LM. Risk of bottle-feeding for rapid weight gain during the first year of life. Arch Pediatr Adolesc Med. (2012) 166:431–6. 10.1001/archpediatrics.2011.1665 22566543

[9] Palmer B. The influence of breastfeeding on the development of the oral cavity: a commentary. J Hum Lactat. (1998) 14:93–8. 10.1177/089033449801400203 9775838

[10] Peres KG Cascaes AM Nascimento GG Victora CG. Effect of breastfeeding on malocclusions: a systematic review and metaanalysis. Acta Paediatr. (2015) 104:54–61. 10.1111/apa.13103 26140303

[11] Sakashita R Inoue N Kamegai T. From milk to solids- a reference standard for the transitional eating process in infants and preschool children in Japan. Eur J Clin Nutr. (2004) 58:643–53. 10.1038/sj.ejcn.1601860 15042133

[12] Harshe Y Aubert B Devezeaux de Lavergne M Modeling the Flow of Infant Formula in a Baby Bottle. Lausanne: Internal Nestlé S.A. (2020).

[13] Pados BF Park J Thoyre SM Estrem H Nix WB. Milk flow rates from bottle nipples used for feeding infants who are hospitalized. Am J Speech Lang Pathol. (2015) 24:671–9. 10.1044/2015_AJSLP-15-0011 26172340 PMC4698468

[14] Nicklaus S Demonteil L Tournier C 8 - Modifying the texture of foods for infants and young children. Kidlington, UK: Modifying Food Texture, Woodhead Publishing (2015). p. 187–222. 10.1016/B978-1-78242-334-8.00008-0

[15] Simione M Loret C Le Révérend B Richburg B Del Valle M Adler M. Differing structural properties of foods affect the development of mandibular control and muscle coordination in infants and young children. Physiol Behav. (2018) 186:62–72. 10.1016/j.physbeh.2018.01.009 29343459 PMC6052439

[16] van der Horst K Deming DM Lesniauskas R Carr BT Reidy KC. Picky eating: associations with child eating characteristics and food intake. Appetite. (2016) 103:286–93. 10.1016/j.appet.2016.04.027 27120094

[17] Doyennette M de Loubens C Déléris I Souchon I Trelea IC. Mechanisms explaining the role of viscosity and post-deglutitive pharyngeal residue on *in vivo* aroma release: a combined experimental and modeling study. Food Chem. (2011) 128:380–90. 10.1016/j.foodchem.2011.03.039 25212145

[18] Emorine M Septier C Andriot I Martin C Salles C Thomas-Danguin T. Combined heterogeneous distribution of salt and aroma in food enhances salt perception. Food Funct. (2015) 6, 1449–59. 10.1039/C4FO01067A 25856503

[19] Feron G Ayed C Qannari EM Courcoux P Laboure H. Understanding aroma release from model cheeses by a statistical multiblock approach on oral processing. PLoS ONE. (2014) 9:e93113. 10.1371/journal.pone.0093113 24691625 PMC3972224

[20] Aubert B Lima A Le Révérend B Biophysical basis of taste modulation by viscous solutions in humans. Food Hydrocoll. (2016) 60:494–9. 10.1016/j.foodhyd.2016.04.018

[21] Lima A Dufauret M le Révérend B Wooster TJ Deconstructing how the various components of emulsion creamers impact salt perception. Food Hydrocoll. (2018) 79:310–8. 10.1016/j.foodhyd.2018.01.005

[22] Burseg KMM Camacho SM Bult JHF. Taste enhancement by pulsatile stimulation is receptor based but independent of receptor type. Chemosens Percept. (2012) 5:179–87. 10.1007/s12078-012-9126-8 22611466 PMC3343238

[23] Thomazo JB Burbidge A Le Révérend B. Frequency-amplitude cross interaction during pulsatile taste delivery using gustometers. Front Neurosci. (2016) 10:562. 10.3389/fnins.2016.00562 28018161 PMC5156721

[24] Le Reverend B Oertling H Gerber B Liquid Dispensing Apparatus. United States Patent Application 20200172316 (2020).

[25] Noort MWJ Bult JHF Stieger M Hamer RJ Saltiness enhancement in bread by inhomogeneous spatial distribution of sodium chloride. J Cereal Sci. (2010) 52:378–86. 10.1016/j.jcs.2010.06.018

[26] Mosca AC Bult JHF Stieger M Effect of spatial distribution of tastants on taste intensity, fluctuation of taste intensity and consumer preference of (semi-)solid food products. Food Qual Prefer. (2013) 28:182–7. 10.1016/j.foodqual.2012.07.003

[27] Upadhyay R Aktar T Chen J. Perception of creaminess in foods. J Text Stud. (2020) 51:375—88. 10.1111/jtxs.12509 32017109

[28] Ranc H Elkhyat A Servais C Mac-Mary S Launay B Humbert P Friction coefficient and wettability of oral mucosal tissue: changes induced by a salivary layer. Colloids Surf A. (2006) 276:155–61. 10.1016/j.colsurfa.2005.10.033

[29] Ranc H Servais C Chauvy P-F Debaud S Mischler S Effect of surface structure on frictional behaviour of a tongue/palate tribological system. Tribol Int. (2006) 39:1518–26. 10.1016/j.triboint.2006.01.017

[30] Bellamy M Godinot N Mischler S Martin N Hartmann C Influence of emulsion composition on lubrication capacity and texture perception. Int J Food Sci Tech. (2009) 44:1939–49. 10.1111/j.1365-2621.2009.02007.x

[31] Engmann J Burbidge AS. Fluid mechanics of eating, swallowing and digestion-overview and perspectives. Food Funct. (2013) 4:443–7. 10.1039/C2FO30184A 23233019

[32] Stokes J Boehm MW Baier S Oral processing, texture and mouthfeel: from rheology to tribology and beyond. Curr Opin Colloid Interf Sci. (2013) 18:349–59. 10.1016/j.cocis.2013.04.010

[33] Garrec D Norton IT The influence of hydrocolloid hydrodynamics on lubrication. Food Hydrocoll. (2012) 26:389–97. 10.1016/j.foodhyd.2011.02.017

[34] Kim JM Wolf F Baiser SK Effect of varying mixing ratio of PDMS on the consistency of the soft-contact Stribeck curve for glycerol solutions. Tribol Int. (2015) 89:46–53. 10.1016/j.triboint.2014.12.010

[35] Pradal C Stokes JR Oral tribology: bridging the gap between physical measurements and sensory experience. Curr Opin Food Sci. (2016) 9:34–41. 10.1016/j.cofs.2016.04.008

[36] Carpenter G Bozorgi S Vladescu S Forte AE Myant C Potineni RV A study of saliva lubrication using a compliant oral mimic. Food Hydrocoll. (2019) 92:10–8. 10.1016/j.foodhyd.2019.01.049

[37] Sarkar A Krop EM. Marrying oral tribology to sensory perception: a systematic review. Curr Opin Food Sci. (2019) 27:64–73. 10.1016/j.cofs.2019.05.007 31903320 PMC6936954

[38] Samaras G Bikos D Vieira J Hartmann C Charalambides M Hardalupas Y Measurement of molten chocolate friction in a simulated tongue-palate system: effect of cocoa solids content and aeration. Curr Res Food Sci. (2020) 3:304–13. 10.1016/j.crfs.2020.10.002 33336192 PMC7733011

[39] Burbidge AS Strassburg J Hartmann C First steps in understanding texture perception in the human mouth as an inverse bio-fluid mechanical problem. AIP Conference Proceedings. In: 15th International Congress on Rheology. (2008). p. 1235–7. 10.1063/1.2964527

[40] Lauga E Pipe CJ Le Révérend B Sensing in the mouth: a model for filiform papillae as strain amplifiers. Front Phys. (2016) 23:35 10.3389/fphy.2016.00035

[41] Rolls BJ Aging and appetite. Nutr Rev. (1992) 50:422–6. 10.1111/j.1753-4887.1992.tb02496.x 1488183

[42] Mowé M Bohmer T Kindt E. Reduced nutritional status in an elderly population (> 70 y) is probable before disease and possibly contributes to the development of disease. Am J Clin Nutr. (1994) 59:317–24. 10.1093/ajcn/59.2.317 8310980

[43] Harris JA Benedict FG A biometric study of the basal metabolism in man. Carnegie Proc Natl Acad Sci U S. (1918) 4:370–3. 10.1073/pnas.4.12.370 PMC1091498 16576330

[44] Frankenfield DC Muth ER Rowe WA. The harris-Benedict studies of human basal metabolism: history and limitations. J Am Dietetic Assoc. (1998) 98:439–45. 10.1016/S0002-8223(98)00100-X 9550168

[45] Rofes L Arreola V Romea M Palomera E Almirall J Cabé M. Pathophysiology of oropharyngeal dysphagia in the frail elderly. Neurogastroenterol Motil. (2010) 22:851–8. 10.1111/j.1365-2982.2010.01521.x 20529208

[46] Clavé P Rofes L Carrión S Ortega O Cabré M Serra-Prat M. Pathophysiology, relevance and natural history of oropharyngeal dysphagia among older people. Nestlé Nutr Inst Workshop Ser. (2012) 72:57–66. 10.1159/000339986 23052001

[47] Serra-Prat M Palomera M Gomez C Sar-Shalom D Saiz A Montoya JG. Oropharyngeal dysphagia as a risk factor for malnutrition and lower respiratory tract infection in independently living older persons: a population-based prospective study. Age and Ageing. (2012) 41:376–81. 10.1093/ageing/afs006 22311895

[48] Ekberg O Hamdy S Woisard V Wuttge-Hannig A Ortega P. Social and psychological burden of dysphagia: its impact on diagnosis and treatment. Dysphagia. (2002) 17:139–46. 10.1007/s00455-001-0113-5 11956839

[49] Burbidge AS Cichero JAY Engmann J Steele CM A day in the life of the fluid bolus: an introduction to fluid mechanics of the oropharyngeal phase of swallowing with particular focus on dysphagia. Appli Rheol. (2016) 64525:1–10. 10.3933/APPLRHEOL-26-64525 PMC8570544 34744553

[50] Marconati M Engmann J Burbidge AS Mathieu V Souchon I Ramaioli M A review of the approaches to predict the ease of swallowing and post-swallow residues. Trends Food Sci Technol. (2019) 86:281–97. 10.1016/j.tifs.2019.02.045

[51] Ketel EC de Wijk RA de Graaf C Stieger M Relating oral physiology and anatomy of consumers varying in age, gender and ethnicity to food oral processing behaviour. Physiol Behav. (2020) 215:112766 10.1016/j.physbeh.2019.112766 31812520

[52] Ketel EC Aguayo-Mendoza MG De Wijk RA De Graaf C Piqueras-Fiszman B Stieger M. Age, gender, ethnicity and eating capability influence oral processing behaviour of liquid, semi-solid and solid foods differently. Food Res Int. (2019) 119:143–51. 10.1016/j.foodres.2019.01.048 30884642

[53] González R Montoya I Cárcel J Review: the use of electromyography on food texture assessment. Food Sci Technol Int. (2001) 7:461–71. 10.1106/NRHT-L39D-HY1Y-8RGB

[54] Gonzalez Espinosa Y Chen J Applications of electromyography (EMG) technique for eating studies. In: Chen J, Engelen L, editors. Food Oral Processing: Fundamentals of Eating and Sensory Perception (West Sussex: Wiley-Blackwell) (2012). p. 289–317. 10.1002/9781444360943.ch13

[55] Funami T Ishihara S Kohyama K Use of electromyography in measuring food texture. In: Lal Dar Y, Light JM, editors. Food Texture Design and Optimization (West Sussex: John Wiley & Sons, Ltd) (2014). p. 283–307. 10.1002/9781118765616.ch11

[56] Foegeding EA Vinyard CJ Essick G Guest S Campbell C Transforming structural breakdown into sensory perception of texture. J Text Stud. (2015) 46:152–70. 10.1111/jtxs.12105

[57] Vinyard CJ Fiszman S Using electromyography as a research tool in food science. Curr Opin Food Sci. (2016) 9:50–5. 10.1016/j.cofs.2016.06.003

[58] Wilson EM Green JR. The development of jaw motion for mastication. Early Hum Deve. (2009) 85:303–11. 10.1016/j.earlhumdev.2008.12.003 19185434 PMC2745715

[59] Koç H Çakir E Vinyard CJ Essick G Daubert CR Drake MA Adaptation of oral processing to the fracture properties of soft solids. J Text Stud. (2014) 45:47–61. 10.1111/jtxs.12051

[60] Le Révérend B Saucy F Moser M Loret C. Adaptation of mastication mechanics and eating behaviour to small differences in food texture. Physiol Behav. (2016) 165:136–45. 10.1016/j.physbeh.2016.07.010 27436795

[61] Wilson A Luck P Woods C Foegeding EA Morgenstern M Comparison of jaw tracking by single video camera with 3D electromagnetic system. J Food Eng. (2016) 190:22–33. 10.1016/j.jfoodeng.2016.06.008

[62] Häggman-Henrikson B Eriksson PO Nordh E Zafar H. Evaluation of skin- versus teeth-attached markers in wireless optoelectronic recordings of chewing movements in man. J Oral Rehabil. (1998) 25:527–34. 10.1046/j.1365-2842.1998.00292.x 9722099

[63] Mathis A Mamidanna P Cury KM Abe T Murthy VN Weygandt Mathis M. DeepLabCut: markerless pose estimation of user-defined body parts with deep learning. Nat Neurosci. (2018) 21:1281–9. 10.1038/s41593-018-0209-y 30127430

[64] Peyron MA Gierczynski I Hartmann C Loret C Dardevet D Martin N. Role of physical bolus properties as sensory inputs in the trigger of swallowing. PLoS ONE. (2011) 6:e21167. 10.1371/journal.pone.0021167 21738616 PMC3124480

[65] Mosca AC van de Velde F Bult JHF Van Boekel M Stieger M Taste enhancement in food gels: effect of fracture properties on oral breakdown, bolus formation and sweetness intensity. Food Hydrocoll. (2015) 43:794–802. 10.1016/j.foodhyd.2014.08.009

[66] Young AK Cheong JN Foster KD Hedderley DI Morgenstern MP James BJ Exploring the links between texture perception and bolus properties throughout oral processing. Part 1: breakdown paths. J Text Stud. (2016) 47:461–73. 10.1111/jtxs.12185

[67] Devezeaux de Lavergne M van de Velde F Stieger M. Bolus matters: the influence of food oral breakdown on dynamic texture perception. Food Funct. (2017) 8:464–80. 10.1039/C6FO01005A 27713955

[68] Bonnet G Batisse C Peyron MA Nicolas E Hennequin M. Which variables should be controlled when measuring the granulometry of a chewed bolus? A systematic review. J Text Stud. (2018) 50:194–216. 10.1111/jtxs.12376 30365162

[69] Allen JE White CJ Leonard RJ Belafsky PC. Prevalence of penetration and aspiration on videofluoroscopy in normal individuals without dysphagia. Otolaryngol Head Neck Surg. (2010) 142:208–13. 10.1016/j.otohns.2009.11.008 20115976

[70] Steele C Grace-Martin K. Reflections on clinical and statistical use of the penetration-aspiration scale. Dysphagia. (2017) 32:601–16. 10.1007/s00455-017-9809-z 28534064 PMC5608795

[71] Mowlavi S Engmann J Burbidge A Lloyd R Hayoun P Le Reverend B. *In vivo* observations and *in vitro* experiments on the oral phase of swallowing of Newtonian and shear-thinning liquids. J Biomech. (2016) 49:3788–95. 10.1016/j.jbiomech.2016.10.011 27823802

[72] Geddes DT Sakalidis VS Ultrasound imaging of breastfeeding—a window to the inside: methodology, normal appearances, and application. J Hum Lactat. (2016) 32:340–9. 10.1177/0890334415626152 26928319

[73] de Wijk RA Wulfert F Prinz JF. Oral processing assessed by M-mode ultrasound imaging varies with food attribute. Physiol Behav. (2006) 89:15–21. 10.1016/j.physbeh.2006.05.021 16820180

[74] Morell P Hernando I Fiszman SM Understanding the relevance of in-mouth food processing. A review of *in vitro* techniques. Trends Food Sci Technol. (2014) 35:18–31. 10.1016/j.tifs.2013.10.005

[75] Gao J Lin S Jin X Wang Y Ying J Dong Z. *In vitro* digestion of bread: how is it influenced by the bolus characteristics? J Text Stud. (2019) 50:257–68. 10.1111/jtxs.12391 30693521

[76] Minekus M Alminger M Alvito P Ballance S Bohn T Bourlieu C. A standardised static *in vitro* digestion method suitable for food - An international consensus. Food Funct. (2014) 5:1113–24. 10.1039/C3FO60702J 24803111

[77] Sarkar A Xu F Lee S. Human saliva and model saliva at bulk to adsorbed phases – similarities and differences. Adv Colloid Interf Sci. (2019) 273:102034. 10.1016/j.cis.2019.102034 31518820

[78] Canon F Neiers F Guichard E Saliva and flavor perception: perspectives. J Agric Food Chem. (2018) 66:7873–9. 10.1021/acs.jafc.8b01998 29962207

[79] Soares S Ferrer-Galego R Brandão E Silva M Mateus N de Freitas V. Contribution of human oral cells to astringency by binding salivary protein/tannin complexes. J Agric Food Chem. (2016) 64:7823–8. 10.1021/acs.jafc.6b02659 27640622

[80] Ployon S Morzel M Belloir C Bonnotte A Bourillot E Briand L. Mechanisms of astringency: structural alteration of the oral mucosal pellicle by dietary tannins and protective effect of bPRPs. Food Chem. (2018) 253:79–87. 10.1016/j.foodchem.2018.01.141 29502847

[81] Xu WL Bronlund JE Potgieter J Foster KD Röhrle O Pullan AJ Review of the human masticatory system and masticatory robotics. Mech Mach Theory. (2008) 43:1353–75. 10.1016/j.mechmachtheory.2008.06.003

[82] Peyron M-A Woda A An update about artificial mastication. Curr Opin Food Sci. (2016) 9:21–8. 10.1016/j.cofs.2016.03.006

[83] Panda S Chen J Benjamin O Development of model mouth for food oral processing studies: present challenges and scopes. Innov Food Sci Emerg Technol. (2020) 66:102524 10.1016/j.ifset.2020.102524

[84] Woda A Mishellany-Dutour A Batier L François O Meunier JP Reynaud B Development and validation of a mastication simulator. J Biomech. (2010) 43:1667–73. 10.1016/j.jbiomech.2010.03.002 20392449

[85] Mishellany-Dutour A Peyron MA Croze J François O Hartmann C Alric M Comparison of food boluses prepared *in vivo* and by the AM2 mastication simulator. Food Qual Pref. (2011) 22:326–31. 10.1016/j.foodqual.2010.12.003

[86] Peyron MA Santé-Lhoutellier V Dardevet D Hennequin M Rémond D François O Addressing various challenges related to food bolus and nutrition with the AM2 mastication simulator. Food Hydrocoll. (2019) 97:105229 10.1016/j.foodhyd.2019.105229

[87] Salles C Tarrega A Mielle P Maratray J Gorria P Liaboeuf J Development of a chewing simulator for food breakdown and the analysis of *in vitro* flavor compound release in a mouth environment. J Food Eng. (2007) 82:189–98. 10.1016/j.jfoodeng.2007.02.008

[88] Yven C Guessasma S Chaunier L Della Valle G Salles C The role of mechanical properties of brittle airy foods on the masticatory performance. J Food Eng. (2010) 101:85–91. 10.1016/j.jfoodeng.2010.06.012

[89] Kristiawan M Della Valle G Réguerre AL Micard V Salles C Artificial oral processing of extruded pea flour snacks. Food Eng Rev. (2020). 10.1007/s12393-020-09220-5. [Epub ahead of print].

[90] Marconati M Pani S Engmann J Burbidge AS Ramaioli M A soft robotic tongue to develop solutions to manage swallowing disorders. arXiv.: arXiv:2003.01194 (2020) 2003:1–19. Available online at: https://arxiv.org/abs/2003.01194

[91] Andablo-Reyes E Bryant M Neville A Hyde P Sarkar R Francis M. 3D biomimetic tongue-emulating surfaces for tribological applications. ACS Appl Mater Interf . (2020) 12:49371–85. 10.1021/acsami.0c12925 33105986 PMC7645869

[92] Chen FJ Dirven S Xu WL Bronlund J Li XN Pullan A Review of the swallowing system and process for a biologically mimicking swallowing robot. Mechatronics. (2012) 22:556–67. 10.1016/j.mechatronics.2012.02.005

[93] Qazi WM Stading M *In vitro* models for simulating swallowing. In: Ekberg O, editor. Dysphagia: Diagnosis and Treatment. Cham: Springer International Publishing (2019). p. 549–62. 10.1007/174_2017_116

[94] Strassburg J Burbidge A Delgado A Hartmann C. Geometrical resolution limits and detection mechanisms in the oral cavity. J Biomech. (2007) 40:3533–40. 10.1016/j.jbiomech.2007.04.012 17976630

[95] Strassburg J Burbidge A Hartmann C Identification of tactile mechanisms for the evaluation of object sizes during texture perception. Food Qual Pref. (2009) 20:329–34. 10.1016/j.foodqual.2009.02.004

[96] Le Révérend B Hartmann C. Numerical modeling of human mastication, a simplistic view to design foods adapted to mastication abilities. Physiol Behav. (2014) 124, 61–4. 10.1016/j.physbeh.2013.10.012 24471180

[97] Skamniotis CG Elliott M Charalambides MN. Computer simulations of food oral processing to engineer teeth cleaning. Nat Commun. (2019) 10:3571. 10.1038/s41467-019-11288-5 31395864 PMC6687884

[98] Harrison SM Cleary PW Eyres G Sinnott MD Lundin L. Challenges in computational modelling of food breakdown and flavour release. Food Funct. (2014) 5:2792–805. 10.1039/C4FO00786G 25277842

[99] Harrison SM Cleary PW Towards modelling of fluid flow and food breakage by the teeth in the oral cavity using smoothed particle hydrodynamics (SPH). Eur Food Res Technol. (2014) 238:185–215. 10.1007/s00217-013-2077-8

[100] Hartmann C Siegrist M Consumer perception and behaviour regarding susta inable protein consumption: a systematic review. Trends Food Sci Technol. (2017) 61:11–25. 10.1016/j.tifs.2016.12.006

[101] Bull SP Hong Y Khutoryanskiy VV Parker JK Faka M Methven L. Whey protein mouth drying influenced by thermal denaturation. Food Qual Pref. (2019 56:233–40. 10.1016/j.foodqual.2016.03.008 28260840 PMC5310118

[102] Bolhuis DP Forde CG Application of food texture to moderate oral processing behaviors and energy intake. Trends Food Sci Technol. (2020) 106:445–56. 10.1016/j.tifs.2020.10.021

[103] van Eck A Stieger M Oral processing behavior, sensory perception and intake of composite foods. Trends Food Sci Technol. (2020) 106:219–31. 10.1016/j.tifs.2020.10.008

